# Lingonberry Improves Non-Alcoholic Fatty Liver Disease by Reducing Hepatic Lipid Accumulation, Oxidative Stress and Inflammatory Response

**DOI:** 10.3390/antiox10040565

**Published:** 2021-04-06

**Authors:** Susara Madduma Hewage, Suvira Prashar, Karmin O, Yaw L. Siow

**Affiliations:** 1 St. Boniface Hospital Research Centre, Winnipeg, MB R2H 2A6, Canada; smaddumahewage@sbrc.ca (S.M.H.); sprashar@sbrc.ca (S.P.); 2 Department of Physiology & Pathophysiology, University of Manitoba, Winnipeg, MB R3E 0J9, Canada; 3 Agriculture and Agri-Food Canada, St. Boniface Hospital Research Centre, Winnipeg, MB R2H 2A6, Canada; 4 Department of Animal Science, University of Manitoba, Winnipeg, MB R3T 2N2, Canada

**Keywords:** fatty liver, high-fat diet, lipids, lingonberry, oxidative stress, Nrf2, glutathione, inflammation

## Abstract

Non-alcoholic fatty liver disease (NAFLD) is the most common chronic liver disease globally and there is a pressing need for effective treatment. Lipotoxicity and oxidative stress are the important mediators in NAFLD pathogenesis. Lingonberry (*Vaccinium vitis-idaea* L.) is rich in anthocyanins that have antioxidant and anti-inflammatory properties. The present study investigated the effect of lingonberry supplementation on liver injury in C57BL/6J male mice fed a high-fat diet (HFD) for 12 weeks. Mice fed HFD displayed liver injury with steatosis, increased lipid peroxidation and inflammatory cytokine expression in the liver as compared to mice fed a control diet. Lingonberry supplementation for 12 weeks alleviated HFD-induced liver injury, attenuated hepatic lipid accumulation, and inflammatory cytokine expression. Lingonberry supplementation inhibited the expression of sterol regulatory element-binding protein-1c (SREBP-1c) and acetyl-CoA carboxylase-1 (AAC-1) as well as activated AMP-activated protein kinase (AMPK) in the liver. It also decreased HFD-induced hepatic oxidative stress and aggregation of inflammatory foci. This was associated with a restoration of nuclear factor erythroid 2–related factor 2 (Nrf2) and glutathione level in the liver. These results suggest that lingonberry supplementation can protect against HFD-induced liver injury partly through attenuation of hepatic lipid accumulation, oxidative stress, and inflammatory response.

## 1. Introduction

Non-alcoholic fatty liver disease (NAFLD) is a major cause of chronic liver injury worldwide [1,2]. It is defined as the hepatic accumulation of excess fat (>5% of hepatocytes) in people who drink little or no alcohol [2,3]. The broad spectrum of NAFLD ranges from simple steatosis (fatty liver) to non-alcoholic steatohepatitis (NASH). NASH is the state of steatosis with hepatic inflammation and ballooning, which can progress to cirrhosis and/or hepatocellular carcinoma (HCC) [3]. The prevalence of NAFLD in general populations has increased up to 25% worldwide [1,2,4]. This is presumptively due to a strong association of NAFLD with metabolic syndrome as NAFLD patients often develop dyslipidemia, hyperglycemia, insulin resistance, and hypertension [5,6]. According to the “two-hit hypothesis” and “multiple-hit hypothesis”, oxidative stress and hepatic inflammation resulted from hepatic free fatty acid overload and *de novo* lipogenesis promote the progression of simple steatosis to advanced liver injury [7,8,9]. 

Obesity or consumption of a high-fat diet (HFD) can lead to an elevation of plasma lipids and glucose, which, in turn, stimulate hepatic lipid accumulation and lipogenesis [10]. Studies conducted in rodent models of obesity and diabetes suggest that increased hepatic lipogenesis in NAFLD is mediated through the activation of sterol regulatory element-binding protein-1c (SREBP-1c) [11,12]. Excess fatty acids in hepatocytes increase the mitochondrial workload and elevate the production of reactive oxygen species (ROS), which promote macromolecule modification such as lipid peroxidation and compromises antioxidant defense in NAFLD [13,14]. ROS and the by-products of lipid peroxidation such as malondialdehyde and 4-hydroxynonenal may activate inflammatory response [15]. Glutathione (*γ*-l-glutamyl-l-cysteinyl-glycine) is the most potent antioxidant that is synthesized and distributed mainly by the liver [16]. It plays a crucial role in protecting cells from oxidative damage and maintaining redox balance [17]. The balance between reduced (GSH) and oxidized (GSSG) glutathione indicates the redox potential, with lower GSH/GSSG ratios suggesting oxidative stress [18]. NAFLD is associated with low levels of plasma and hepatic GSH [19,20]. The nuclear factor, erythroid 2-related factor 2 (Nrf2), is a key transcription factor in regulating antioxidant and xenobiotic stress responses [21]. Under normal conditions, Nrf2 is kept in the cytosol by its main inhibitory regulatory protein Kelch-like ECH-associated protein 1 (Keap1) [21]. However, during oxidative stress, the Keap1/Nrf2 complex is dissociated. The liberated Nrf2 is translocated into the nucleus, where it binds the Nrf2-targeted antioxidant response element (ARE) and upregulates the transcription of target genes encoding proteins that are involved in antioxidant defense. These include glutathione-synthesizing enzymes glutamate–cysteine ligase (catalytic subunit Gclc, modifier subunit Gclm) and glutathione synthetase (GS) [16,22]. 

With the increased prevalence and lack of treatment options for NAFLD, there is an urgent need to seek effective alternatives. Lifestyle and dietary habits are major risk factors as well as protective factors in the development and progression of NAFLD [23]. Diets rich in fruits and vegetables are among the recommended lifestyle modifications to decrease the risk of metabolic syndrome and degenerative diseases [23]. Lingonberry (*Vaccinium vitis-idaea* L.) is a small reddish color berry that grows in North America and Eurasia throughout the Northern Hemisphere, which contains a significantly higher anthocyanin content than the commonly consumed berries [24,25]. Lingonberry has been widely used in traditional Scandinavian diets not only due to its appealing color to the food, but also its richness in vitamins and polyphenols [26]. In Quebec, Canada, the Cree of Eeyou Istchee community use lingonberry as a remedy for symptoms of diabetes [27]. Recent studies including ours have demonstrated that lingonberry anthocyanins have antioxidant, anti-inflammatory, and anti-diabetic properties [28,29,30]. A previous study evaluated the metabolic effects of various berries including lingonberry in HFD-fed mice [31]. Supplementation of lingonberry (20% *w/w*) significantly decreased body fat content, body weight gain, hepatic lipid accumulation, and plasminogen activator inhibitor-1 as well as improved glucose homeostasis in HFD-fed mice [31]. Another study demonstrated that lingonberry supplementation attenuated glycemia and insulin resistance in muscle cells and improved hepatic lipid profile through the AMPK/Akt pathways in HFD-fed mice [30]. Although the effects of lingonberry supplementation on the reduction of body fat and body weight gain as well as the improvement of lipid and glucose metabolism have been reported in obese or diabetic animal models [30,31,32], the role of lingonberry in hepatic oxidative stress associated with NAFLD has yet to be examined. The present study investigated the effect and mechanisms of lingonberry supplementation at a lower dose (5% *w/w*) on hepatic steatosis, oxidative stress, and inflammatory response in a mouse model with HFD-induced fatty liver injury.

## 2. Materials and Methods

### 2.1. Animal Model 

The C57BL/6J male mice, acquired from the University of Manitoba Central Animal Care Services, were housed two per cage in a temperature and humidity-controlled room with a 12 h dark–12 h light cycle. There were three experimental groups of mice (6 weeks of age, *n* = 6 in each group) that were fed the following diet: (1) Control (D12450J, Research Diets Inc., Brunswick, NJ, USA) diet consisted of 11% kcal fat, 18% kcal protein, and 71% kcal carbohydrate, (2) HFD (D12492) consisted of 62% kcal fat, 18% kcal protein, and 20% kcal carbohydrate, or (3) HFD supplemented with (5% *w/w*) Manitoba lingonberry (*Vaccinium vitis-idaea* L.) (D17022206). The source of fat in HFD was derived from lard (90%) and soybean oil (10%). Lingonberry was harvested in Manitoba, Canada, and immediately frozen at −20 °C. In a pilot study, HFD was supplemented with 1% (*w/w*) or 5% (*w/w*) lingonberry. Supplementation of lingonberry at 5% (*w/w*) reduced plasma and liver lipid levels in HFD fed mice. In the present study, the freeze-dried berry powder was provided to Research Diet Inc. and was incorporated at 5% (*w/w*) into the HFD diet. The animals were fed the above diets *ad libitum* for 12 weeks, body weight and feed intake were recorded. The average feed intake was assessed by manual weighing of feed before and after a feeding period every second day. After the 12-week experimental period, blood and liver were collected from the mice following sacrifice. Plasma triglyceride, total cholesterol, alanine transaminase (ALT), and aspartate transaminase (AST) were measured using a Cobas C111 Analyzer (Roche, Risch-Rotkreuz, Switzerland). All procedures were performed in accordance with the Guide to the Care and Use of Experimental Animals published by the Canadian Council on Animal Care and approved by the University of Manitoba Protocol Management and Review Committee (Protocol No. B2015-072). 

### 2.2. Biochemical Assays

Malondialdehyde (MDA), a stable product of lipid peroxidation, was assessed in the liver using the thiobarbituric acid reactive substances (TBARS) as previously described [33]. The reduced and oxidized glutathiones, GSH and GSSG, were also measured using previously described procedures [34]. Hepatic lipids were extracted from liver tissue using the Folch method [33,35]. Hepatic total cholesterol and triglyceride levels in the lipid extracts were determined using commercial kits according to the manufacturer’s instructions (SEKISUI Diagnostics, Burlington, MA, USA) [34].

### 2.3. Measurement of mRNA Expression 

From the liver tissues that were preserved in RNA*later* (Thermo Fisher Scientific, Waltham, MA, USA), total RNA was extracted with QIAzol reagent (Qiagen, Hilden, Germany) [29]. Relative mRNA expression of glutamate–cysteine ligase catalytic subunit (*Gclc*), glutamate–cysteine ligase modifier subunit (*Gclm*), glutathione synthetase (*GS*), sterol regulatory element-binding protein-1c (*SREBP-1c*), acetyl-CoA carboxylase-1 (*ACC-1*), interleukin-6 (*IL-6*), monocyte chemoattractant protein-1 (*MCP-1*), and tumor necrosis factor-*α* (*TNF-α*) were measured using a StepOne Plus Real-Time qPCR (RT-qPCR) system (Applied Biosystems, Foster City, CA, USA), using previously described protocol [29]. Total RNA was extracted according to the procedure for the isolation of RNA as described by Chomczynski and Mackey [36]. All the data were analyzed using the comparative C_T_ method [37] with gene expression level normalized to that of the housekeeping gene *β-Actin*. Primer sequences used for the RT-qPCR were shown in Table 1. 

### 2.4. Western Immunoblotting 

Total proteins were extracted from mouse liver tissues in lysis buffer [20 mM Tris pH 7.4, 150 mM NaCl, 1 mM EGTA, 1 mM EDTA, 2.5 mM sodium pyrophosphate, 1 mM β-glycerophosphate, 1 mM sodium orthovanadate, 2.1 μM leupeptin, 1 mM PMSF, and 1% (*v/v*) Triton X-100] and were separated by electrophoresis in a 10% or 12% SDS-polyacrylamide gel as previously described [29,38]. Following electrophoresis and electrotransfer, the membranes were probed with rabbit anti-Gclc monoclonal antibody (1:1000), rabbit anti-Gclm monoclonal antibody (1:1000), or rabbit anti-GS monoclonal antibody (1:1000), which were purchased from Abcam, Cambridge, UK. To determine the relative amount of phosphorylated or total AMP-activated protein kinase-alpha (AMPK-*α*) in the liver, the membranes were probed with rabbit anti-phospho-AMPK-*α* monoclonal antibody (1:1000) or rabbit anti-AMPK-*α* monoclonal antibody (1:1000) that were purchased from Cell Signaling Technology (Danvers, MA, USA) [39]. To ensure equal protein loading, the same membrane was probed with rabbit anti-*β*-Actin primary antibody (1:5000, Cell Signaling Technology). Nuclear proteins were prepared as previously described [29,38]. Nuclear Nrf2 and SREBP-1c proteins were identified by using rabbit anti-Nrf2 monoclonal antibodies (1:1000, Abcam) and rabbit anti-SREBP-1c monoclonal antibodies (1:1000, Santa Cruz Biotechnology, Inc., Dallas, TX, USA), respectively. To ensure equal loading of nuclear proteins, the same membrane was probed with rabbit anti-Lamin B1 (nuclear envelope marker) polyclonal primary antibody (1:2000, Abcam). In all immunoblots, HRP-conjugated anti-rabbit IgG secondary antibodies (Cell Signaling Technology) were used. The protein bands were visualized by using ECL detection system (Millipore Ltd., Burlington, MA, USA) and quantified using Quantity One software version 4.6.8 for Windows (Bio-Rad, CA, USA).

### 2.5. Histological Staining 

Paraffin-sectioned livers (5 μm thickness) were stained with hematoxylin and eosin (H&E) to evaluate the morphological changes [40]. Another set of paraffin-embedded sections were immunostained as previously described [29], using rat anti-F4/80 antibody (1:100 dilution, MCA497, Bio-Rad), followed by biotinylated goat anti-rat IgG (1:200, Dako, Glostrup, Denmark) and streptavidin-horse radish peroxidase (HRP) conjugate (Zymed Laboratories, Inc., San Francisco, CA, USA). These slides were counterstained with Mayer’s hematoxylin. For the negative controls, normal rat IgG was used as primary antibodies. To stain for neutral lipids, frozen liver samples were cut into 10 μm thick sections using a Leica CM1850 UV Cryostat (Wetzlar, Germany). The frozen sections were stained with Oil Red O to visualize lipid droplets in the liver [40]. All the images were captured using an Olympus BX43 Upright Light Microscope (Olympus Corp., Tokyo, Japan) equipped with a Q-color 3 digital camera and analyzed using Image-Pro Plus 7.0 (Media Cybernetics, Rockville, MD, USA).

### 2.6. Statistical Analysis

The results were analyzed using a two-tailed Student’s t-test and expressed as mean ± standard error (SE). All statistical analyses were performed using ProStat Version 6 software (Poly Software International, Pearl River, NY, USA). A *p*-value of less than 0.05 was considered statistically significant.

## 3. Results

### 3.1. Effect of HFD Feeding and Lingonberry Supplementation on Body Weight and Liver Injury 

**Figure 1:**
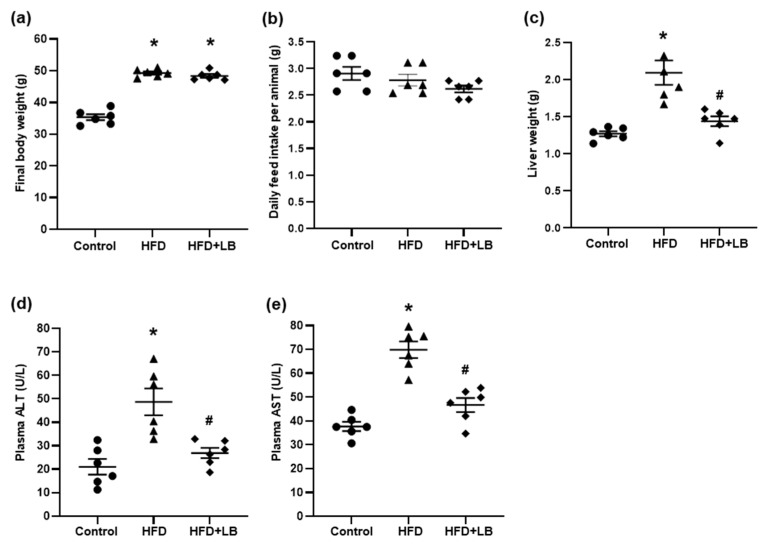
Effect of HFD feeding and lingonberry supplementation on body weight and liver injury. Mice were fed a control diet, high-fat diet (HFD) or HFD supplemented with lingonberry (HFD+LB) for 12 weeks. (**a**) Bodyweight was measured at the end of the feeding period. (**b**) Feed intake was measured. At the end of 12 weeks, (**c**) liver weight, plasma (**d**) alanine transaminase (ALT), and (**e**) aspartate transaminase (AST) were measured. The results are expressed as mean ± SE (*n* = 6). * *p* < 0.05 when compared with the value obtained from the control group. ^#^ *p* < 0.05 when compared with the value obtained from the HFD group.

At the start of the experimental study, the average body weight of the mice ranged from 22 to 24 g. Following a 12-week HFD feeding period, there was a significant elevation of body weight in these mice when compared to the mice fed a control diet (Figure 1a). With supplementation of lingonberry over the 12-week study period, there was no change in the body weight gain induced by HFD. There was no significant difference in the feed intake among groups (Figure 1b). The liver weight of HFD-fed mice was significantly increased compared to that of mice fed a control diet and HFD-fed mice with lingonberry supplementation (Figure 1c). The HFD feeding induced liver injury, as indicated by a significant elevation of plasma ALT and AST (Figure 1d,e) levels. Supplementation of HFD with lingonberry resulted in a significant decline in plasma ALT and AST (Figure 1d,e). 

### 3.2. Effect of HFD Feeding and Lingonberry Supplementation on Plasma and Liver Lipids 

**Figure 2:**
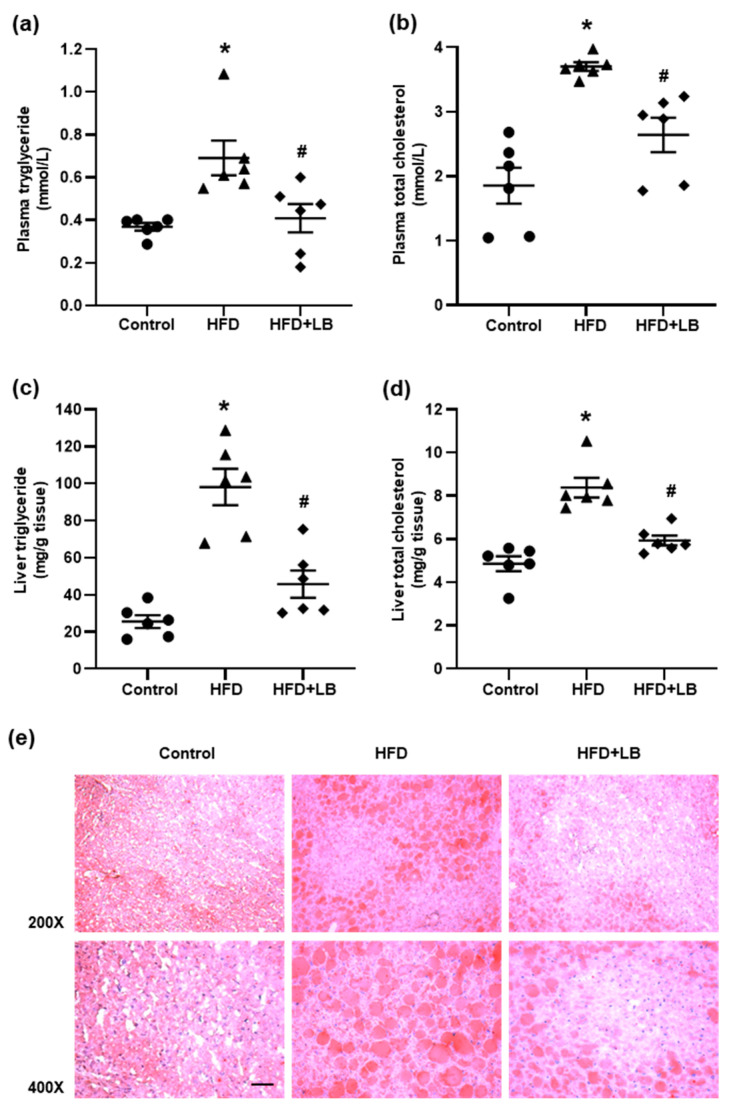
Effects of HFD feeding and lingonberry supplementation on plasma and liver lipid profiles. Mice were fed a control diet, high-fat diet (HFD) or HFD supplemented with lingonberry (HFD+LB) for 12 weeks. (**a**) Plasma triglyceride, (**b**) plasma total cholesterol, (**c**) liver triglyceride, and (**d**) liver total cholesterol levels were measured. (**e**) Frozen sections of liver tissues were stained with Oil Red O staining for neutral lipids (scale bar = 100 μm). The results are expressed as the means ± SE (*n* = 6). * *p* < 0.05 when compared with the value obtained from the control group. ^#^ *p* < 0.05 when compared with the value obtained from the HFD group.

HFD feeding resulted in a significant elevation of triglyceride and total cholesterol levels in the plasma (Figure 2a,b). Lingonberry supplementation reduced the plasma lipid levels in mice fed HFD. Mice fed HFD had significantly higher triglyceride and total cholesterol levels in the liver compared to the mice fed a control diet (Figure 2c,d). Supplementation of lingonberry significantly reduced hepatic accumulation of triglyceride and cholesterol (Figure 2c,d). Liver tissue was also examined with Oil Red O staining (Figure 2e). There were increased lipid vacuoles/droplets in the liver of mice fed HFD compared to the mice fed a control diet. Mice fed HFD supplemented with lingonberry exhibited fewer and smaller hepatic lipid vacuoles/droplets compared to the HFD fed mice (Figure 2e).

### 3.3. Effect of HFD Feeding and Lingonberry Supplementation on the Indicators of De Novo Lipogenesis 

**Figure 3:**
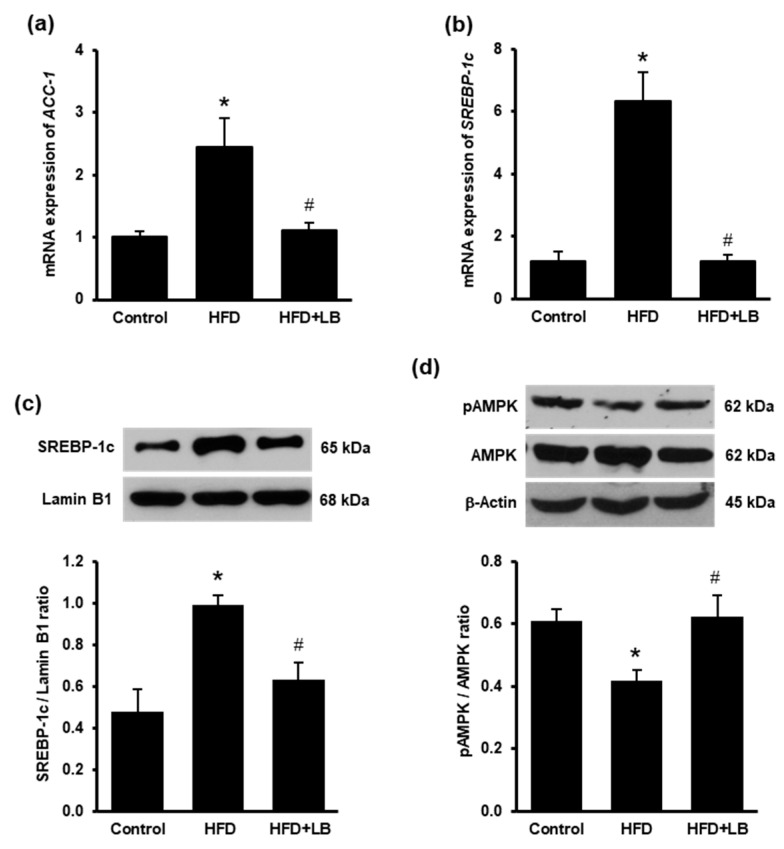
Effects of HFD feeding and lingonberry supplementation on the indicators of *de novo* lipogenesis in the liver. Mice were fed a control diet, high-fat diet (HFD) or HFD supplemented with lingonberry (HFD+LB) for 12 weeks. Relative mRNA expression of (**a**) acetyl-CoA carboxylase-1 (*ACC-1*) and (**b**) sterol regulatory element-binding protein-1c (*SREBP-1c*) were measured in the liver. (**c**) The SREBP-1c protein in the nucleus was determined by Western immunoblotting analysis. (**d**) Phosphorylated AMPK (pAMPK) and total AMPK were determined by Western immunoblotting analysis. The results are expressed as the means ± SE (*n* = 4 to 6). * *p* < 0.05 when compared with the value obtained from the control group. ^#^ *p* < 0.05 when compared with the value obtained from the HFD group.

To assess the liver *de novo* lipogenesis, mRNA expression of acetyl-CoA carboxylase-1 *(ACC-1)* and sterol regulatory element-binding protein-1c *(SREBP-1c)* were measured. HFD feeding significantly elevated *ACC-1* and *SREBP-1c* mRNA in the liver (Figure 3a,b). Supplementation of lingonberry reduced hepatic *ACC-1* and *SREBP-1c* mRNA expression (Figure 3a,b). Western immunoblotting analysis revealed that HFD feeding increased the nuclear protein level of SREBP-1c (Figure 3c). Lingonberry supplementation significantly reduced the nuclear protein level of SREBP-1c (Figure 3c). Phosphorylation of AMPK was assessed by detecting phosphorylated AMPK (pAMPK) relative to total AMPK protein levels in the liver (Figure 3d). HFD feeding resulted in a significant reduction of pAMPK and pAMPK/AMPK ratio compared to the control diet-fed animals. Lingonberry supplementation restored pAMPK level and pAMPK/AMPK ratio (Figure 3d).

### 3.4. Effect of HFD Feeding and Lingonberry Supplementation on Liver Lipid Peroxidation and Glutathione Levels

**Figure 4:**
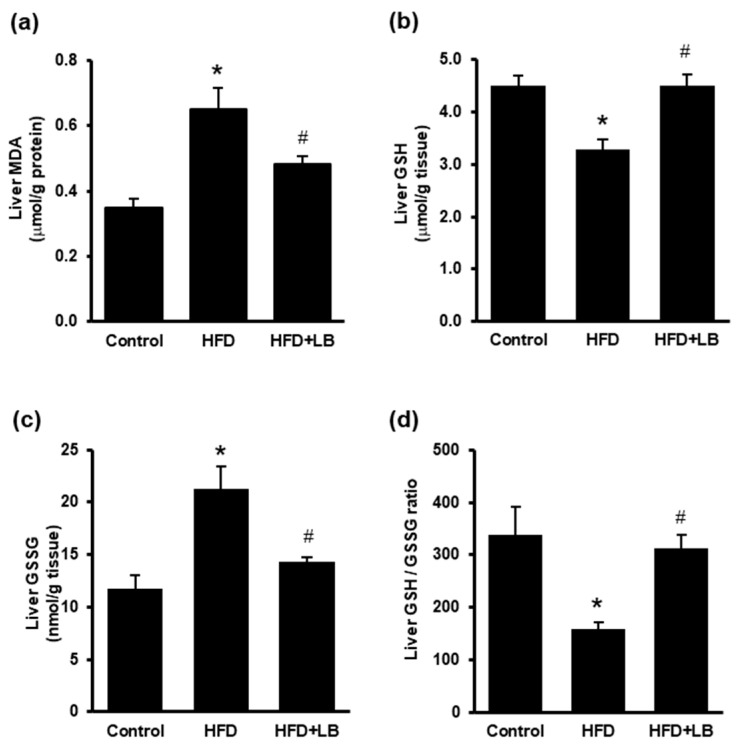
Effect of HFD feeding and lingonberry supplementation on liver lipid peroxidation and glutathione level. Mice were fed a control diet, high-fat diet (HFD) or HFD supplemented with lingonberry (HFD+LB) for 12 weeks. (**a**) Malondialdehyde (MDA) levels, (**b**) reduced glutathione (GSH) levels, (**c**) oxidized glutathione levels (GSSG), and (**d**) GSH/GSSG ratio were measured in the liver. The results are expressed as the means ± SE (*n* = 5 to 6). * *p* < 0.05 when compared with the value obtained from the control group. ^#^ *p* < 0.05 when compared with the value obtained from the HFD group.

HFD feeding resulted in a significant elevation of hepatic malondialdehyde (MDA) levels and a decrease in reduced glutathione (GSH) in the liver tissues (Figure 4a,b). Lingonberry supplementation reduced HFD-induced MDA levels and restored GSH levels (Figure 4a,b). Hepatic oxidized glutathione (GSSG) level was significantly increased and the ratio of GSH/GSSG was decreased in mice fed a HFD (Figure 4c,d). Lingonberry supplementation reduced the hepatic GSSG level and restored the GSH/GSSG ratio (Figure 4c,d).

### 3.5. Effect of HFD Feeding and Lingonberry Supplementation on Glutathione Synthesis

**Figure 5:**
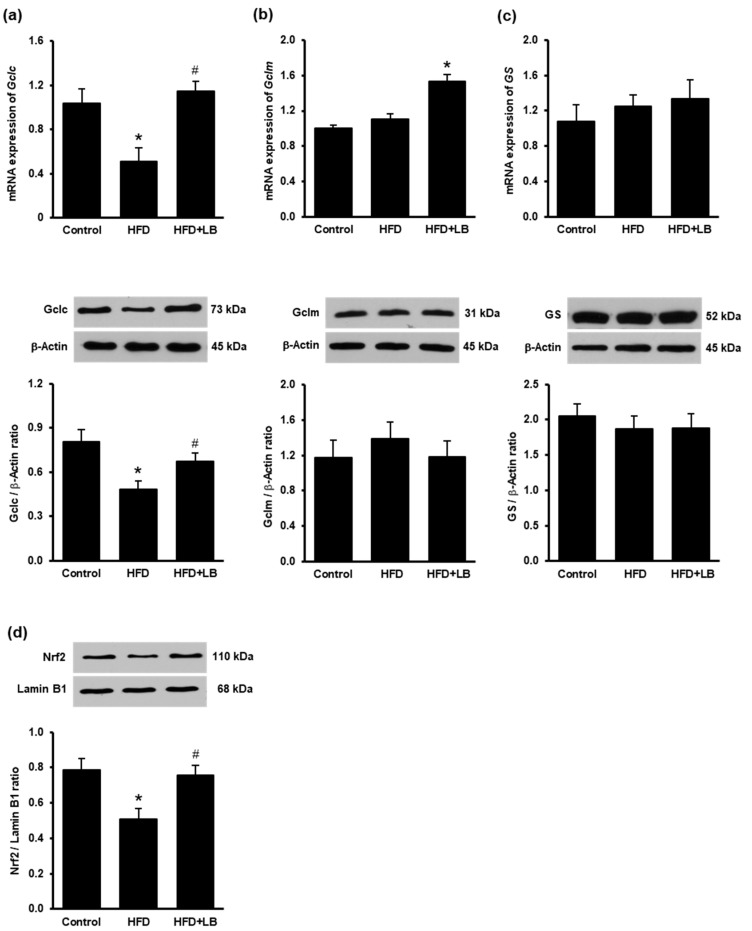
Effect of HFD feeding and lingonberry supplementation on glutathione synthesis. Mice were fed a control diet, high-fat diet (HFD) or HFD supplemented with lingonberry (HFD+LB) for 12 weeks. Relative mRNA and protein levels of (**a**) glutamate–cysteine ligase catalytic subunit (Gclc), (**b**) glutamate–cysteine ligase modifier subunit (Gclm), and (**c**) glutathione synthetase (GS) were measured in the liver. (**d**) The Nrf2 protein in the nucleus was determined by Western immunoblotting analysis. The results are expressed as the means ± SE (*n* = 4 to 6). * *p* < 0.05 when compared with the value obtained from the control group. ^#^ *p* < 0.05 when compared with the value obtained from the HFD group.

Gene and protein levels of glutathione synthesizing enzymes glutamate–cysteine ligase (catalytic subunit Gclc, modifier subunit Gclm) and glutathione synthetase were measured in the liver. Gene and protein levels of Gclc was significantly reduced in the liver during the HFD feeding (Figure 5a). Supplementing HFD with lingonberry increased the expression of Gclc in the liver (Figure 5a). Although HFD feeding did not significantly affect Gclm and gluthatione synthetase expression (Figure 5b,c), lingonberry supplementation increased Gclm mRNA levels in the liver (Figure 5b). Mice fed HFD had a significantly low level of nuclear Nrf2 protein compared to the control group (Figure 5d). Lingonberry supplementation restored nuclear Nrf2 protein level in the liver (Figure 5d).

### 3.6. Effect of HFD Feeding and Lingonberry Supplementation on Hepatic Inflammation

**Figure 6:**
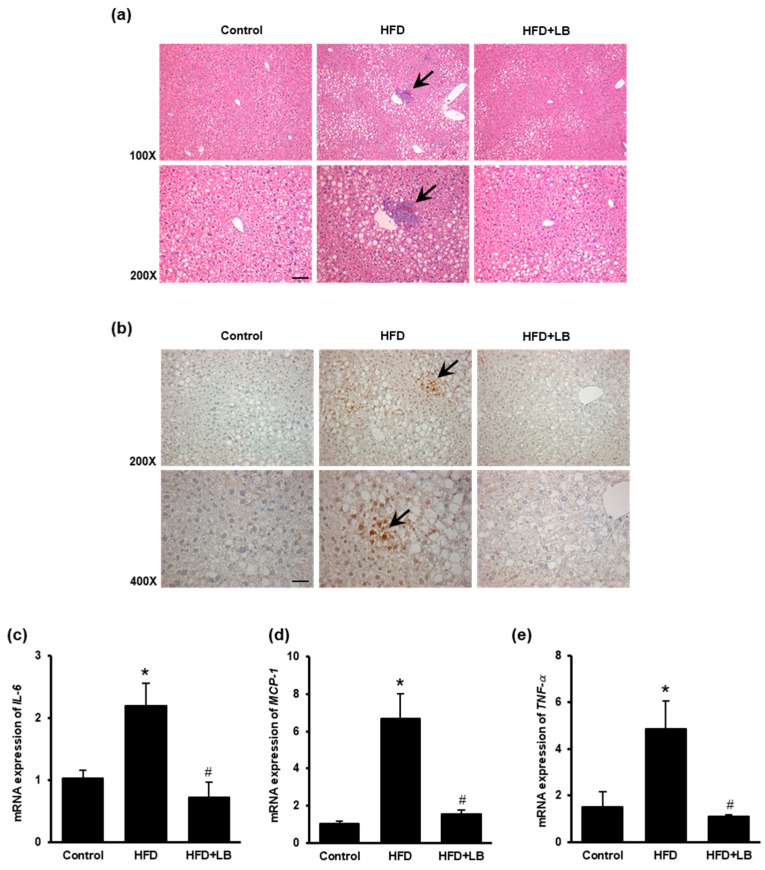
Effects of HFD feeding and lingonberry supplementation on liver inflammation. Mice were fed a control diet, high-fat diet (HFD) or HFD supplemented with lingonberry (HFD+LB) for 12 weeks. (**a**) Paraffin sections of the liver tissues were stained with hematoxylin and eosin (H&E) to examine liver histology. Arrows point to inflammatory foci (scale bar = 100 μm). (**b**) Paraffin sections of the liver tissues were stained with anti-F4/80 antibodies to detect macrophages in the inflamed areas. Arrows point to macrophages (scale bar = 100 μm). Relative mRNA expression of (**c**) interleukin-6 (*IL-6*), (**d**) monocyte chemoattractant protein-1 (*MCP-1*), and (**e**) tumor necrosis factor-*α* (*TNF-α*) were measured in the liver. The results are expressed as the means ± SE (*n* = 5–6). * *p* < 0.05 when compared with the value obtained from the control group. ^#^ *p* < 0.05 when compared with the value obtained from the HFD group.

The H&E staining revealed a noticeable deposition of inflammatory foci in the liver of mice fed HFD (Figure 6a). Such inflammatory foci were not observed in mice fed a control diet or with lingonberry supplementation. Immunohistochemical staining of the liver tissue sections with anti-F4/80 antibody (Macrophages specific marker) revealed increased infiltration of macrophages in the liver of mice fed HFD (Figure 6b). To further confirm hepatic inflammation, gene expression of inflammatory cytokines, *IL-6*, *MCP-1*, and *TNF-α* were assessed. HFD feeding significantly elevated hepatic *IL-6*, *MCP-1,* and *TNF-α*-mRNA levels (Figure 6c–e). Supplementation of lingonberry reduced *IL-6*, *MCP-1,* and *TNF-α*-mRNA expression (Figure 6c–e). 

## 4. Discussion

Nutritional intervention is emerging as a promising management strategy for NAFLD and obesity. Hepatic lipid accumulation, oxidative stress, and inflammatory response play a central role in the pathogenesis of NAFLD [3,7]. In the present study, mice fed HFD for 12 weeks developed features of fatty liver including hepatic steatosis, oxidative stress, and increased inflammatory cytokine expression with increased body weight gain and impaired liver function. Supplementation with lingonberry protected HFD-induced liver injury potentially through (1) attenuation of hepatic lipid accumulation; (2) reduction of oxidative stress through the restoration of Nrf2/glutathione synthesis; and (3) inhibition of inflammatory cytokine expression.

Obesity or HFD feeding can elevate plasma lipid levels and cause hepatic lipid overload. Accumulation of fat in hepatocytes higher than 5% is referred to as fatty liver, a hallmark of NAFLD [3]. Elevation of plasma and hepatic lipids induces insulin resistance, which stimulates hepatic *de novo* lipogenesis, one of the mechanisms for hepatic lipid accumulation in NAFLD [10]. In the present study, we observed a significant reduction of triglyceride and cholesterol levels in the plasma and liver tissue in mice supplemented with lingonberry. Such a lipid-lowering effect by lingonberry supplementation was independent of body weight change. Lipogenesis plays an important role in hepatic lipid accumulation and elevation of blood lipid levels through secretion of very-low-density lipoprotein (VLDL). SREBP-1c is a key transcription factor that regulates lipogenesis through upregulating gene expression of enzymes responsible for lipogenesis including ACC-1, the rate-limiting enzyme of lipogenesis [11,12,41]. We observed that HFD-induced hepatic lipid accumulation was accompanied by the upregulation of SREBP-1c and ACC-1 expression. Lingonberry supplementation attenuated HFD-induced SREBP-1c and ACC-1 expression as well as reduced plasma lipid levels and improved fatty liver. Furthermore, we observed that lingonberry supplementation restored hepatic AMPK activation that was attenuated by HFD feeding. The AMPK plays a central role in energy sensing and hepatic metabolism. With widespread control over a variety of metabolic cascades, AMPK regulation is an important mediator in NAFLD, in which both energy homeostasis and metabolic function are perturbed [42]. One of the mechanisms of AMPK action is through phosphorylation of SREBP-1c at Ser372, hence inhibits proteolytic cleavages and nuclear translocation of SREBP-1c, which in turn, inhibits hepatic lipogenesis [43]. Previous studies reported that lingonberry supplementation at a higher dose (20%, *w/w*) significantly decreased body weight and epididymal fat content [31,32], which, in turn, might contribute to a reduction of hepatic lipid accumulation. Results from the present study suggested that attenuation of SREBP-1c and restoration of AMPK by lingonberry supplementation at a lower dose might account for a reduction of hepatic lipid accumulation, which was independent of weight gain change. 

Chronic oxidative stress and inflammation trigger NAFLD progression to NASH [15,44]. Elevated ROS levels can affect the expression and activity of enzymes that are involved in lipid metabolism. It has been reported that increased ROS impairs fatty acid oxidation and promotes fatty acid esterification into triglycerides that are stored in lipid droplets [13]. ROS at excessive amounts also disrupts insulin signaling, elicits an immune response, promotes macromolecule modification, and compromises antioxidant defense in NAFLD [13,45]. Malondialdehyde (MDA) is formed during lipid peroxidation, which is one of the toxic and mutagenic aldehydes [46]. Increased oxidative stress can lead to the redox-dependent dysregulation of hepatic metabolism and function. The “multiple hit” hypothesis suggests multiple insults acting together on genetically predisposed subjects to induce NAFLD and provides a comprehensive explanation of NAFLD pathogenesis [8,9]. In the present study, lingonberry supplementation attenuated HFD-induced oxidative stress by reducing hepatic MDA formation. Glutathione is the most abundant non-enzymatic antioxidant that plays a crucial role in the detoxification and antioxidant defense in the liver [16,17]. A decline of glutathione has been reported in NAFLD patients [19,20]. In the present study, there was a significant reduction of glutathione levels and the GSH/GSSG ratio, indicative of oxidative stress, in the liver of HFD-fed mice. Lingonberry supplementation effectively restored both the hepatic glutathione level and the GSH/GSSG ratio. Glutathione is synthesized by glutamate–cysteine ligase and glutathione synthase. Glutamate–cysteine ligase, the enzyme that catalyzes the rate-limiting step in glutathione biosynthesis, comprises a catalytic subunit (Gclc) and a modifier subunit (Gclm) [16,17]. It was plausible that increased expression of glutamate–cysteine ligase (Gclc) might have contributed to increased glutathione synthesis in the liver of mice by lingonberry supplementation. 

The transcriptional factor Nrf2 is a key transcription factor involved in cellular responses against oxidative stress. It regulates the expression of antioxidant enzymes including those responsible for glutathione synthesis. In the present study, HFD feeding reduced the levels of GSH and nuclear Nrf2 protein in the liver. It was reported that genetic ablation of Nrf2 in primary mouse embryo fibroblast cells and liver reduced the expression of glutamate–cysteine ligase and intracellular glutathione [47]. Under physiological conditions, Nrf2 remains in the cytoplasm by binding to Keap1, an endogenous inhibitor that mediates the rapid ubiquitination and subsequent degradation of Nrf2 by proteasomes [21]. Under oxidative stress conditions, Keap1/Nrf2 complex dissociates, and Nrf2 translocates into the nucleus, where it binds to the antioxidant response element (ARE) and activates the transcription of several antioxidative enzymes including glutathione-synthesizing enzymes [16,22]. Our results suggested that lingonberry supplementation attenuated HFD-induced oxidative stress through increased Nrf2/glutathione antioxidant defense. Such a beneficial effect of lingonberry might be attributed to its high content of anthocyanins. Our recent study has identified cyanidin-3-galactoside (C3Gal), cyanidin-3-arabinoside (C3Ara), and cyanidin-3-glucoside (C3Glu) as the three anthocyanins found in lingonberry, which possess high antioxidant potentials [48]. It was reported that C3Glu could activate the Nrf2/ARE pathway in human umbilical vein endothelial cells challenged with TNF-α [49]. In another study, C3Glu activated Nrf2 signaling and reduced palmitic acid-induced lipotoxicity in intestinal epithelial cells [50]. Future studies are warranted to identify the contribution of individual anthocyanins or other active ingredients in lingonberry to its antioxidant action. 

The present study was performed using a well-established HFD feeding animal model with features that resembled characteristics of NAFLD. Our results, for the first time, demonstrated that lingonberry supplementation at 5% (*w/w*) had a hepatic protective effect against HFD-induced liver injury through lipid-lowering, antioxidant and anti-inflammatory response. Such a hepatic protective effect of lingonberry was independent of body weight changes. Our results suggested that the antioxidant effect of lingonberry might be mediated through the restoration of Nrf2 and glutathione biosynthesis. However, the active ingredients in lingonberry that may contribute to hepatic protective effect remain to be identified in future studies. 

## 5. Conclusions

**Figure 7:**
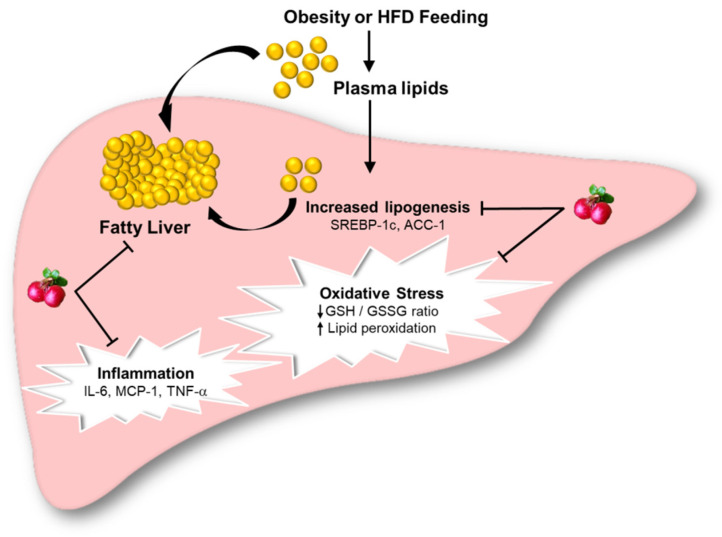
Graphical illustration of lingonberries protective effect on HFD-induced fatty liver and oxidative stress. Abbreviations: HFD = High-fat diet; SREBP-1c = sterol regulatory element-binding protein-1c; ACC-1 = acetyl-CoA carboxylase-1; GSH = reduced glutathione; GSSG = oxidized glutathione; *IL-6* = interleukin-6; MCP-1 = monocyte chemoattractant protein-1; TNF-*α* = tumor necrosis factor-*α*.

In conclusion, our results demonstrate that chronic consumption of HFD causes hepatic lipid accumulation, oxidative stress, and inflammation, resulting in liver damage (Figure 7). Lingonberry supplementation confers protection against HFD-induced liver injury through improving hepatic steatosis, attenuating oxidative stress, and inflammatory response (Figure 7). Our results suggest that regulation of hepatic lipid synthesis and activation of the Nrf2 signaling pathway may contribute to the beneficial effect of lingonberry in the context of NAFLD, which is independent of body weight changes. In view that there are no effective pharmacological agents currently approved for NAFLD, consumption of lingonberry may serve as a potential alternative for the management of NAFLD.

## Figures and Tables

**Table 1:** Primer sequences used for the RT-qPCR.

Gene	Forward Primer (5′–3′)	Reverse Primer (5′–3′)	Accession Number	Size (bp)
*Gclc*	GGGGTGACGAGGTGGAGTA	GTTGGGGTTTGTCCTCTCCC	NM_010295.2	125
*Gclm*	CGAGGAGCTTCGAGACTGTAT	ACTGCATGGGACATGGTACA	NM_008129.4	114
*GS*	CACTGGGTCGTACCCAAGC	ATACGTCACCACTCGCTCGT	NM_001291111.1	98
*SREBP-1c*	GGAGCCATGGATTGCACATT	GGCCCGGGAAGTCACTGT	XM_006532716.4	70
*ACC-1*	CGGACCTTTGAAGATTTTGTGAGG	GCTTTATTCTGCTGGTGTAACTCTC	XM_036156218.1	223
*IL-6*	GACTGATGCTGGTGACAACC	GCCATTGCACAACTCTTTTC	NM_001314054.1	170
*MCP-1*	AGGTCCCTGTCATGCTTCTG	GCTGCTGGTGATCCTCTTGT	NM_011333.3	167
*TNF-α*	GTCCCCAAAGGGATGAGAAG	GCTCCTCCACTTGGTGGTTT	NM_001278601.1	93
*β-Actin*	GATCAAGATCATTGCTCCTCCT	AGGGTGTAAAACGCAGCTCA	XM_030254057.1	183

## Data Availability

Data is contained within the article.
